# Genomic Characterization of a Tetracycline-Resistant Strain of *Brochothrix thermosphacta* Highlights Plasmids Partially Shared between Various Strains

**DOI:** 10.3390/genes14091731

**Published:** 2023-08-30

**Authors:** Antony T. Vincent, Romain P. Bergeron, Laurie C. Piché, David Prado, Linda Saucier

**Affiliations:** 1 Département des Sciences Animales, Faculté des Sciences de L’agriculture et de L’alimentation, Université Laval, Quebec City, QC G1V 0A6, Canada; 2 Institut de Biologie Intégrative et des Systèmes, Université Laval, Quebec City, QC G1V 0A6, Canada; 3 Institut sur la Nutrition et les Aliments Fonctionnels, Faculté des Sciences de L’agriculture et de L’alimentation, Université Laval, Quebec City, QC G1V 0A6, Canada; 4 Site de Bourg-end-Bresse, IUT Lyon 1 Site de Bourg-en-Bresse, 01000 Bourg-en-Bresse, France

**Keywords:** antibiotic resistance, *Brochothrix thermosphacta*, food, plasmids, *tet*(L), tetracycline

## Abstract

The Gram-positive bacterium *Brochothrix thermosphacta* is a spoilage agent commonly found on meat products. While the *tet*(L) gene, which confers resistance to tetracycline, has been identified in certain strains of *B. thermosphacta*, only a limited number of studies have investigated this gene and its potential presence on mobile DNA elements. This study aims to analyze the tetracycline-resistant strain *B. thermosphacta* BT469 at the genomic level to gain insight into the molecular determinants responsible for this resistance. Three plasmids have been identified in the strain: pBT469-1, which contains a *tetR* gene; pBT469-2, which harbours the *tet*(L) gene responsible for tetracycline resistance; and pBT469-3, which carries genes encoding for a thioredoxin and a phospholipase A2. Homology searches among sequences in public databases have revealed that the plasmid pBT469-2 is currently unique to the BT469 strain. However, the pBT469-1 plasmid is also found in three other strains of *B. thermosphacta*. Notably, sequences similar to pBT469-1 and pBT469-2 were also found in other bacterial genera, suggesting that these plasmids may be part of a diverse family present in several bacterial genera. Interestingly, sequences of various strains of *B. thermosphacta* show a high level of similarity with pBT469-3, suggesting that variants of this plasmid could be frequently found in this bacterium.

## 1. Introduction

Food waste is a major issue recognized by the Food and Agriculture Organization [1]. Despite efforts throughout the value chain, meat, because of its high nutrient density, is by nature perishable and conducive to microbial growth [2]. Contamination with spoilage agents can cause physical and organoleptic properties of the meat to change, making it undesirable for consumption [3]. Several bacteria, such as *Pseudomonas* sp., *Leuconostoc*, *Acinetobacter*, and various lactic acid bacteria and *Enterobacteriaceae*, can spoil meats [4]. It is well-known and recently exemplified that the presence of spoilage bacteria depends on the meat storage temperature. On meat wrapped in commercial polyethylene stored at approximately 4 °C—the recommended storage temperature for meat—the two dominant bacteria were *Pseudomonas* sp. and *Brochothrix thermosphacta* [5].

The Gram-positive bacterium *B. thermosphacta* is facultatively anaerobic, making it an excellent candidate for spoiling meats because it can grow on meat in air and faulty vacuum storage, where small amounts of oxygen are still present. Commonly found on meat, this bacterium appears to be resident in slaughterhouses [6], although the original ecological niche remains uncertain. In the presence of oxygen, it can ferment glucose to produce volatile organic compounds, mainly acetoin [6], a molecule involved in the development of a cheesy odor in spoiled meat [7].

Plasmids are self-replicating and usually extrachromosomal DNA elements. Although considered non-essential, plasmids can confer important genes to their host bacteria, notably for virulence, metabolism, or antibiotic resistance [8]. In *B. thermosphacta*, few plasmids have been characterized. In 2017, Stanborough et al. found a *tet*(L) gene, causing tetracycline resistance, on a potential plasmid sequence of strain Bth-7811 [9]. Subsequently, Illikoud et al. reported the genomic sequences of four strains of *B. thermosphacta* and identified four small plasmids [6]. Finally, in 2019, Höll et al. deposited the sequences of two *B. thermosphacta* plasmids (pL21564-1 and pL21572-1) as part of a study on bacteria in modified-atmosphere packaged poultry meat [10]. However, these plasmids have not been subjected to exhaustive analysis.

In the present study, the genomic analysis of *B. thermosphacta* BT469, resistant to tetracycline, has revealed the presence of three small plasmids, including one with a *tet*(L) gene. The characterization of these three plasmids highlighted that *B. thermosphacta* could have a relatively diversified repertoire of small plasmids, and that some of these could belong to a family with variants present in other bacterial genera.

## 2. Materials and Methods

### 2.1. BT469 Isolation and Tetracycline Resistance

The *B. thermosphacta* BT469 strain was isolated in the province of Quebec (Canada) in 1998 on STAA medium at 25 °C from vacuum-packed smoked cooked ham. The strain was stored lyophilized until recently when it was recultured in HIB medium (Oxoid Inc, Ottawa, ON, Canada) at 25 °C, and then frozen at −80 °C in 20% glycerol as a cryoprotectant.

To assess the minimum inhibitory concentration (MIC) of tetracycline, the strains BT469 and DSM 20171 were thawed on HIB-agar medium and incubated for approximately 24 h at 25 °C. An isolated colony was picked and mixed with 4 mL of liquid HIB medium and incubated for approximatively 4 h until a satisfactory optical density (O.D._600nm_ ≈ 0.8) was reached. Next, 1 mL of this culture was used to produce a bacterial lawn on HIB-agar medium. A strip of tetracycline antibiotic (Liofilchem^®^, Waltham, MA, USA) was aseptically deposited on the bacterial film in the center of the petri dish. Finally, the agar plate was incubated for 24 h at 25 °C before reading the MIC value according to the manufacturer’s instructions. The experiment was carried out in duplicate for each strain.

### 2.2. Sequencing and Bioinformatics Analyses

Total DNA of strain BT469 was extracted with QIAamp PowerFecal Pro DNA Kit (QIAGEN, Toronto, ON, Canada) from a culture grown on HIB-agar medium and incubated for 24 h at 25 °C and following the manufacturer’s instructions. The extracted DNA was quantified by fluorescence using PicoGreen kit (Invitrogen, Waltham, MA, USA). Whole genome shotgun library preparation and sequencing was performed at the Plateforme d’Analyses Génomiques of the Institut de Biologie Intégrative et des Systèmes (IBIS, Université Laval, Québec, QC, Canada). Briefly, a whole genome shotgun barcoded library was prepared with 50 ng of DNA using 1/5 reaction volumes of the NEBNext UltraII FS (Ipswich, MA, USA) following the manufacturer’s instructions. An ECHO 525 acoustic liquid handler (Beckman Coulter Life Sciences, Indianapolis, IN, USA) was used for precise distribution of the reagents. The barcoded library was then sequenced by a MiSeq apparatus (Illumina, San Diego, CA, USA).

Sequencing reads were filtered with fastp version 0.23.2 [11], and de novo assembled by SPAdes version 3.14.1 [12] through shovill version 1.1.0 (https://github.com/tseemann/shovill, accessed on 1 June 2023). Genomic sequences were annotated with Bakta version 1.8.1 [13], and antibiotic resistance genes were identified with AMRFinderPlus version 3.11.2 [14]. The presence of a CRISPR-Cas system was predicted by the CRISPRCasFinder web server [15].

Plasmid sequences were determined by subtracting chromosomal sequences with CONTIGUator version 2.7 [16] using the *B. thermosphacta* BI strain chromosome sequence (GenBank: CP023483.1) as a template. Subsequently, the circularity of the contigs (redundant ends) was checked by comparing the first 500 and the last 500 bp of each sequence with the water tool of emboss suite version 6.6.0.0 [17]. Plasmid sequences were visualized with Artemis version 18.1.0 [18] and their maps drawn with DNAPlotter version 18.1.0 [19]. A search for homologous sequences was done by BLASTn for each of the plasmid sequences against two databases, nr/nt, which contains closed sequences, and wgs, which includes all the draft sequences (several contigs). Sequence comparisons were also performed with EasyFig version 2.2.2 [20].

Plasmid copy number of strain BT469 was inferred by mapping sequencing reads to plasmid and chromosomal sequences with Bowtie version 2.5.1 [21] and samtools version 1.17 [22]. The sequencing depth was calculated with qualimap version 2.3 [23]. The copy number was calculated for each of the plasmids by dividing its sequencing depth value by that of the chromosomal sequences.

The whole genome shotgun sequences of BT469, and the annotated sequences of plasmids pBT469-1, pBT469-2, and pBT469-3 were deposited in DDBJ/ENA/GenBank under the accession JAUPXA000000000, OR354882, OR354883, and OR354884, respectively.

## 3. Results

A strain of *B. thermosphacta*, BT469, was found to have tetracycline resistance during antibiotic screening of a strain collection isolated in the late 1990s and early 2000s. The MIC of tetracycline for this strain was 48 µg/mL, whereas the MIC for the type-strain, DSM 20171, was 0.25 µg/mL.

**Figure 1:**
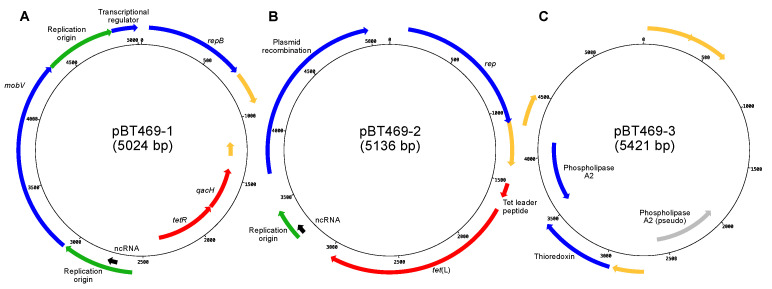
Maps of plasmids (**A**) pBT469-1, (**B**) pBT469-2, and (**C**) pBT469-3. The red, yellow, and blue arrows, respectively, represent the genes coding for proteins involved in resistance to antibiotics (or quaternary ammonium compounds), with a hypothetical function, or for another function (for example, the maintenance of the plasmid or its mobilization). Green, black, and gray arrows represent regulatory regions, non-coding RNAs (ncRNAs), and pseudogenes, respectively.

The DNA of strain BT469 was sequenced at high throughput to determine the molecular mechanism responsible for tetracycline resistance. Assembly and annotation allowed the reconstruction of 39 contigs totaling 2,663,328 bp with 2549 CDSs, 78 tRNAs, and a CRISPR array predicted to be type 1-C. A search for antibiotic resistance genes revealed a *tet*(L) gene, providing resistance to tetracycline [24]. Analysis of circular sequences detected the sequences of three small plasmids of 5024 bp, 5136 bp, and 5421 bp, here named pBT469-1, pBT469-2, and pBT469-3, respectively. The plasmid pBT469-1 has a *tetR* gene (Figure 1A), which encodes a protein, TetR, known to regulate *tet* genes [25]. It also contains the *qacH* gene, which encodes resistance to disinfectants based on quaternary ammonium compounds [26]. Also, on pBT469-1, automatic annotation revealed two origins of replications located on either side of the *mobV* gene, involved in the mobility of the plasmid. Plasmid pBT469-2 is directly responsible for the resistance to tetracycline because it possesses the *tet*(L) gene (Figure 1B) and a gene coding for a leader peptide that regulates the expression of *tet*(L) [27]. A putative origin of replication was identified with a BLAST analysis against the DoriC database [28]. Plasmid pBT469-3 has a gene coding for a thioredoxin and a gene coding for a phospholipase A2 (Figure 1C). A second gene encoding phospholipase A2 is also present but is predicted to be a pseudogene because of a frameshift. No origin of replication was predicted for pBT469-3. Notably, two other sequences have redundant sequences at their ends and, therefore, are potentially circular: contig_5 of 127,238 bp and contig_10 of 7043 bp. However, the sequences have no clear plasmid genes such as those involved in mobilization, conjugation, or replication, or a high identity with known plasmid sequences. Therefore, these sequences were not further investigated in the present study.

Automatic annotation of the plasmid sequences revealed the presence of a non-coding RNA within the plasmids pBT469-1 and pBT469-2. Although the two plasmid sequences are not homologous, both RNA sequences have 86.44% sequence identity over 86% of their sequence length, based on a BLASTn analysis. This is the only region where pBT469-1 and pBT469-2 share sequence identity. According to the RNA-family database (Rfam), the two RNAs are from the same family, which corresponds to *Bacillus*-plasmid RNA (Rfam: RF01691). This RNA family is commonly found in *Bacillus* and *Lactobacillus* plasmids and is thought to play a role in regulating the copy number of plasmids. As indicated from Rfam, the structure of the RNAs of this family is similar to the R1162-like plasmid antisense RNA that regulates plasmid copy number [29]. By comparing the coverage values of the pBT469-1 and pBT469-2 sequences with those of the chromosomal sequences (considered to be at one copy per cell), it was possible to estimate that pBT469-1 and pBT469-2 have approximately four to six copies per cell. Therefore, if the RNAs of pBT469-1 and pBT469-2 are indeed involved in the regulation of plasmid copy number, the sequence differences between the RNAs do not seem to affect their function. RNA structure prediction shows similar structures and also supports a common function (Supplementary Figure S1). The plasmid pBT469-3 is predicted to be present in a single copy per cell and its mode of replication remains unknown.

**Figure 2:**
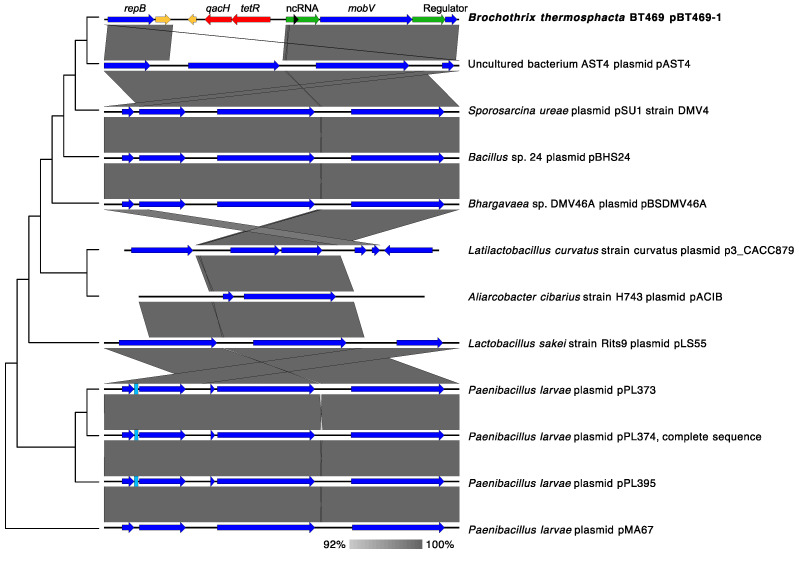
Alignment of the pBT469-1 sequence against the closest homologous sequences in the nr/nt according to a phylogenetic analysis based on BLASTn. Homologous regions between sequences are shown in gray. The red, yellow, and blue arrows represent, respectively, the genes coding for proteins involved in resistance to antibiotics (or quaternary ammonium compounds), with a hypothetical function, or for another function (for example, the maintenance of the plasmid or its mobilization). Green and black arrows represent regulatory regions and non-coding RNAs (ncRNAs), respectively.

A BLASTn search with the NCBI nr/nt database revealed that the pBT469-1 sequence shares similar regions with several sequences, but none corresponds exactly to the plasmid pBT469-1 (Supplementary Figure S2). These sequences come from very different bacterial genera, even focusing on the closest sequences according to BLASTn (Figure 2 and Supplementary Figure S3). This suggests that pBT469-1 may belong to a group of related plasmids present in different bacterial genera. By comparing the sequence of pBT469-1 to all *B. thermosphacta* sequences in the wgs database, three strains (BSAS1 3, TAP 175, and Bth-7818) were found to contain pBT469-1 in their genome (Supplementary Figure S4). The strains BSAS1 3, TAP 175, and Bth-7818 were isolated from a beef slaughterhouse, fresh chicken leg, and aerobically stored beef, respectively [9,30]. This demonstrates that pBT469-1 can be found in strains from different sources.

**Figure 3:**
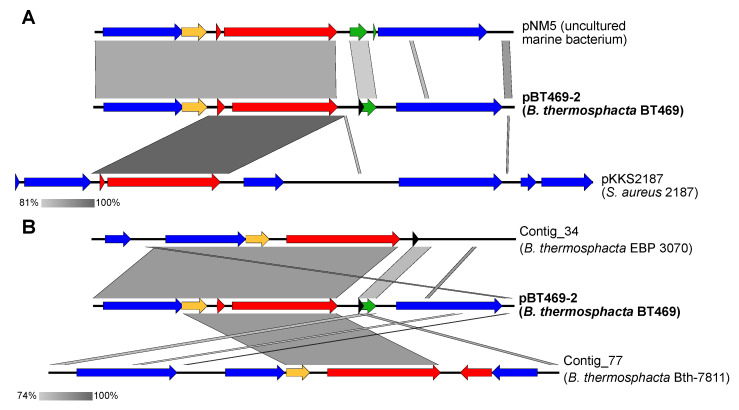
Alignment of the sequence of pBT469-2 against the homologous sequences of (**A**) the nr/nt and (**B**) wgs databases for *B. thermosphacta*. Homologous regions between sequences are shown in gray. The red, yellow, and blue arrows represent, respectively, the genes coding for proteins involved in resistance to antibiotics, with a hypothetical function, or for another function (for example, the maintenance of the plasmid or its mobilization). Green and black arrows represent regulatory regions and non-coding RNAs (ncRNAs), respectively.

BLASTn of the plasmid pBT469-2 sequence against the nr/nt database revealed similar regions in the sequence of plasmid pNM5 from an uncultured marine bacterium (Figure 3A). Although the sequence of the plasmid pNM5 has the highest level of identity with the sequence of pBT469-2, several plasmids and chromosomal sequences of the bacterium *Staphylococcus aureus* show homology for the *tet*(L) gene (Supplementary Figure S5). The sequence of plasmid pKKS2187 exhibiting the second highest level of identity with plasmid pBT469-2 (Figure 3A), after the sequence of pNM5. A BLASTn search between the pBT469-2 sequence and the *B. thermosphacta* sequences from the NCBI wgs database revealed only two *B. thermosphacta* sequences (EBP 3070 and Bth-7811) with regions homologous to the pBT469-2 sequence (Figure 3B). The EBP 3070 strain was isolated from spoiled smoked salmon [30], while the Bth-7811 strain comes from beef stored under aerobic conditions [9]. In both cases, the regions have the *tet*(L) gene, but the complete sequence of the plasmid pBT469-2 was not found.

**Figure 4:**
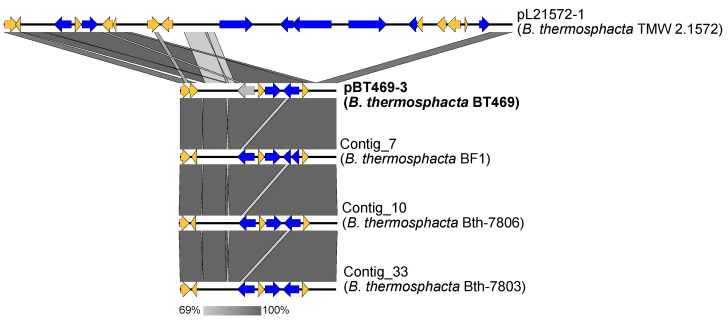
Alignment of the sequence of pBT469-3 against the homologous sequences from the nr/nt and wgs (for *B. thermosphacta*) databases. Homologous regions between sequences are shown in gray. The yellow and blue arrows represent, respectively, the genes coding for proteins with a hypothetical function, or for another function (for example, the maintenance of the plasmid or its mobilization). Gray arrows represent pseudogenes.

Interestingly, the sequence of plasmid pBT469-3 was found almost entirely in the sequence of plasmid pL21572-1 available in the nr/nt database (Figure 4). Plasmid pL21572-1 comes from the *B. thermosphacta* TMW 2.1572 strain, isolated from poultry meat [31]. However, the sequence of plasmid pL21572-1 is longer (17,660 bp) than plasmid pBT469-3 (5421 bp). By comparing the sequence of the plasmid pBT469-3 to the *B. thermosphacta* sequences of the wgs database, 10 sequences had a high level of identity with the sequence of pBT469-3 (Supplementary Figure S6). Of these 10 sequences, those originating from strains BF1, Bth-7806, and Bth-7803 were perfectly collinear with the sequence of pBT469-3 (Figure 4). The BF1 strain was isolated from a meat processing facility [31], while the Bth-7806 and Bth-7803 strains were isolated from aerobically stored beef and modified atmosphere packaged beef [9], respectively.

## 4. Discussion

Antibiotic resistance is an increasingly worrying phenomenon in several spheres of human activity [32]. Horizontal transfer of antibiotic resistance genes between bacteria makes them difficult to contain in order to control the spread of antibiotic resistance [33]. Therefore, it is crucial to adopt a global vision (One Health) to better understand the network of interaction between bacteria in the same ecological niche.

Meat products are of great importance in the human diet and are by their nature conducive to promoting microbial growth [2]. Although the *B. thermosphacta* bacterium is not considered a pathogen, it is very often found on meat products and is associated with spoilage [34]. Because of its ubiquitous presence in meat and meat products [35], it is reasonable to believe that it actively participates in the bacterial ecosystem of meat, including in the spread of antibiotic resistance genes.

This study has characterized three plasmids found in *B. thermosphacta* strain BT469. Two of these plasmids, pBT469-1 and pBT469-2, are involved in resistance to tetracycline, and pBT469-1 also confers resistance to quaternary ammonium compounds. The *tet*(L) gene, found on pBT469-2, has already been detected in *B. thermosphacta* [9,30], although this plasmid has not been described in the literature. Tetracycline is one of the most widely used antibiotics globally [36], and it is not surprising to find a resistance gene for this antibiotic. However, given the strict withdrawal time following antibiotic treatment before animals enter the human food chain, generally little or no antibiotic is found in the meat [36]. Therefore, direct selection pressure on the meat must be very low compared with other environments, such as the microbiota of animals. Therefore, selection may occur elsewhere and on other bacteria, and the gene is subsequently transferred to *B. thermosphacta*, making this bacterium a relay point for gene exchange. This hypothesis is particularly plausible in the context that homologous regions of the two plasmids BT469-1 and pBT469-2 have been found in other bacterial genera.

It is interesting that the *tetR* gene, which regulates the activity of *tet* genes, is on pBT469-1 while *tet*(L) is on pBT469-2. It is currently unclear if the two genes act in concert or if they are independent. Because pBT469-1 is found in some strains without pBT469-2, pBT469-1 may be independent of pBT469-2. However, the reverse remains unknown because, for the moment, pBT469-2 has only been found in the BT469 strain. Another interesting feature of pBT469-1 is that the plasmid is predicted to have two origins of replication. Plasmids with several origins of replication have already been listed in the literature, for example, pJD4 of *Neisseria gonorrhoeae*, which encodes a β-lactamase [37].

Plasmid pBT469-3, although not implicated in tetracycline resistance, has a gene coding for a thioredoxin and a gene coding for a phospholipase A2. Thioredoxin participates in the protection against antioxidants and has recently been identified as a promising therapeutic target against pathogens [38]. The production of phospholipase A2 is known in some pathogens, such as *Pseudomonas aeruginosa*, and helps modulate host-pathogen interactions, such as the host immune response [39]. In the case of *B. thermosphacta*, which is not considered pathogenic, the role of phospholipase remains unknown. The plasmid may have been transferred from a pathogenic bacterium or the enzyme has an unknown role that would allow *B. thermosphacta* greater success within its ecological niche. This plasmid is frequently found in strains of *B. thermosphacta*; therefore, it could be advantageous to the strains that possess it. So far, no origin of replication has been identified in pBT469-3. It is possible that the completion of databases and the improvement of bioinformatics methods may in the future help to identify the origin of replication of this plasmid. It is also interesting that pBT469-3 is entirely found in pL21572-1. However, it is not yet clear whether pBT469-3 derives from pL21572-1 by loss of genes or, on the contrary, whether pL21572-1 is derived from a gain of genes in pBT469-3.

The propensity of *B. thermosphacta* to acquire plasmids and to participate in horizontal transfer events remains largely unknown. Interestingly, several phage genes have been detected in the genomic sequences of *B. thermosphacta*, and these genes would constitute a large part of the genomic variability between known strains [9]. However, there is a dichotomy among *B. thermosphacta* strains: some have a CRISPR-Cas system, while others do not. CRISPR-Cas systems allow phage resistance by acquiring phage DNA fragments (spacers) which can serve as a template for recognizing and degrading the phage DNA during subsequent infections [40]. This system has also been shown to work against plasmids by recognizing and degrading plasmid DNA in a similar process as for phage DNA [41]. Interestingly, the BT469 strain has three plasmids, despite the predicted presence of a CRISPR-Cas system, but it is not known if the system is functional. Investigation of more strains would be necessary to determine the propensity of *B. thermosphacta* to acquire genes horizontally and if molecular determinants may promote or restrict horizontal transfers in this bacterium.

## 5. Conclusions

The genomic characterization of the tetracycline-resistant strain BT469 has shed light on three new plasmids in the *B. thermosphacta* bacterium, a meat spoilage agent. The importance of *B. thermosphacta* within its ecological niche remains to be determined. The investigation of the plasmid repertoire of this bacterium will highlight its ecological role and its contribution to horizontal gene transfers. This study suggests that this bacterium may possess plasmids that confer advantages, such as resistance to antibiotics. However, the provenance of these plasmids remains unknown, as does the ability of *B. thermosphacta* to transfer these elements to other bacteria. The increasingly easy access to long-read sequencing technologies (PacBio and Oxford Nanopore), which allow the assembly of complex plasmid sequences [42], will allow a better understanding of the role of DNA elements in pathogens and bacteria responsible for food losses, such as *B. thermosphacta*. It is realistic to believe that it will soon be possible to map the gene exchange network involving *B. thermosphacta*. To do this, it will also be crucial to continue to isolate strains from different sources and geographical locations to have a complete view of its diversity.

## Data Availability

The whole genome shotgun sequences of BT469, and the annotated sequences of plasmids pBT469-1, pBT469-2, and pBT469-3, were deposited in DDBJ/ENA/GenBank under the accession JAUPXA000000000, OR354882, OR354883, and OR354884, respectively.

## Supplementary Materials

The following supporting information can be downloaded at: https://www.mdpi.com/article/10.3390/genes14091731/s1, Figure S1: Prediction of the ncRNA structures for the plasmids pBT469-1 and pBT469-2.; Figure S2: BLASTn result (50 best hits) of the pBT469-1 sequence against the nr/nt database.; Figure S3: Phylogenetic tree generated with BLASTn from the NCBI server.; Figure S4: Alignment of the pBT469-1 sequence against the homologous sequences in the wgs database for *B. thermosphacta*.; Figure S5: BLASTn result (50 best hits) of the pBT469-2 sequence against the nr/nt database.; Figure S6: BLASTn result (50 best hits) of the pBT469-3 sequence against the wgs database (for *B. thermosphacta*).
