# Supplementary material for: Genomic Characterization of a Tetracycline-Resistant Strain of Brochothrix thermosphacta Highlights Plasmids Partially Shared between Various Strains
Source: Genes (Basel). 2023 Aug 30;14(9):1731. doi: 10.3390/genes14091731 (PMC10531132; doi:10.3390/genes14091731)
Supplement: Supplementary file 1 [file genes-14-01731-s001.zip › genes-2562140-supplementary.pdf]

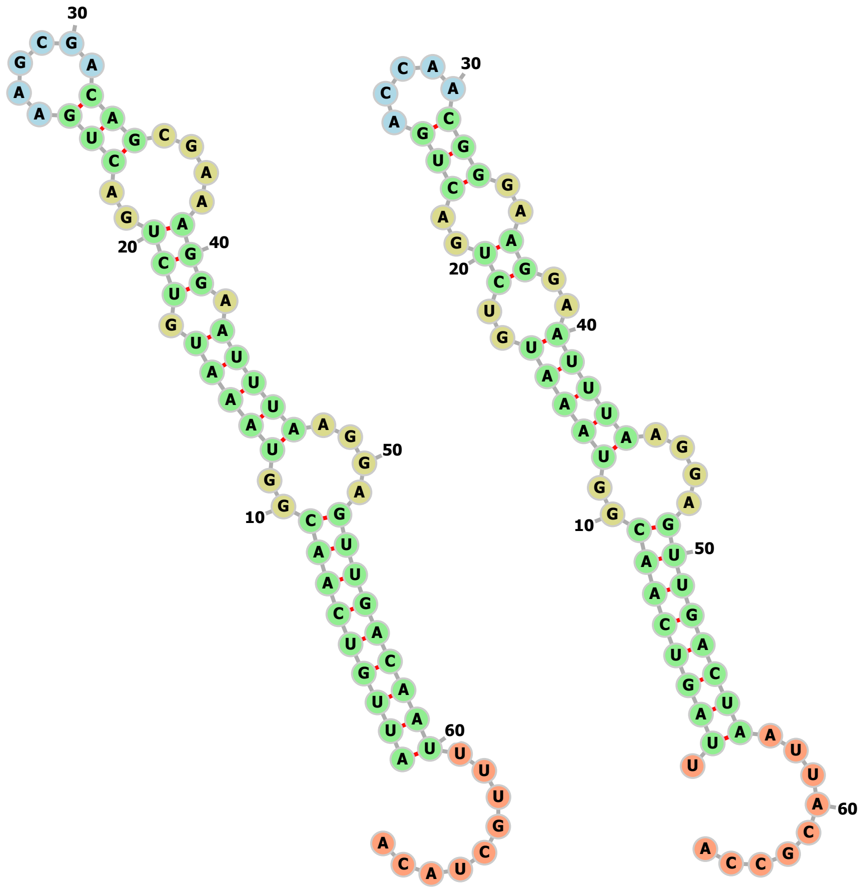

pBT469-1 pBT469-2

Figure S1. Prediction of the ncRNA structures for the plasmids pBT469-1 and pBT469-2. The different RNA structures are colored: green, gtems (canonical helices); yellow, interior loops; blue, hairpin loops; orange, 5' and 3' unpaired regions.

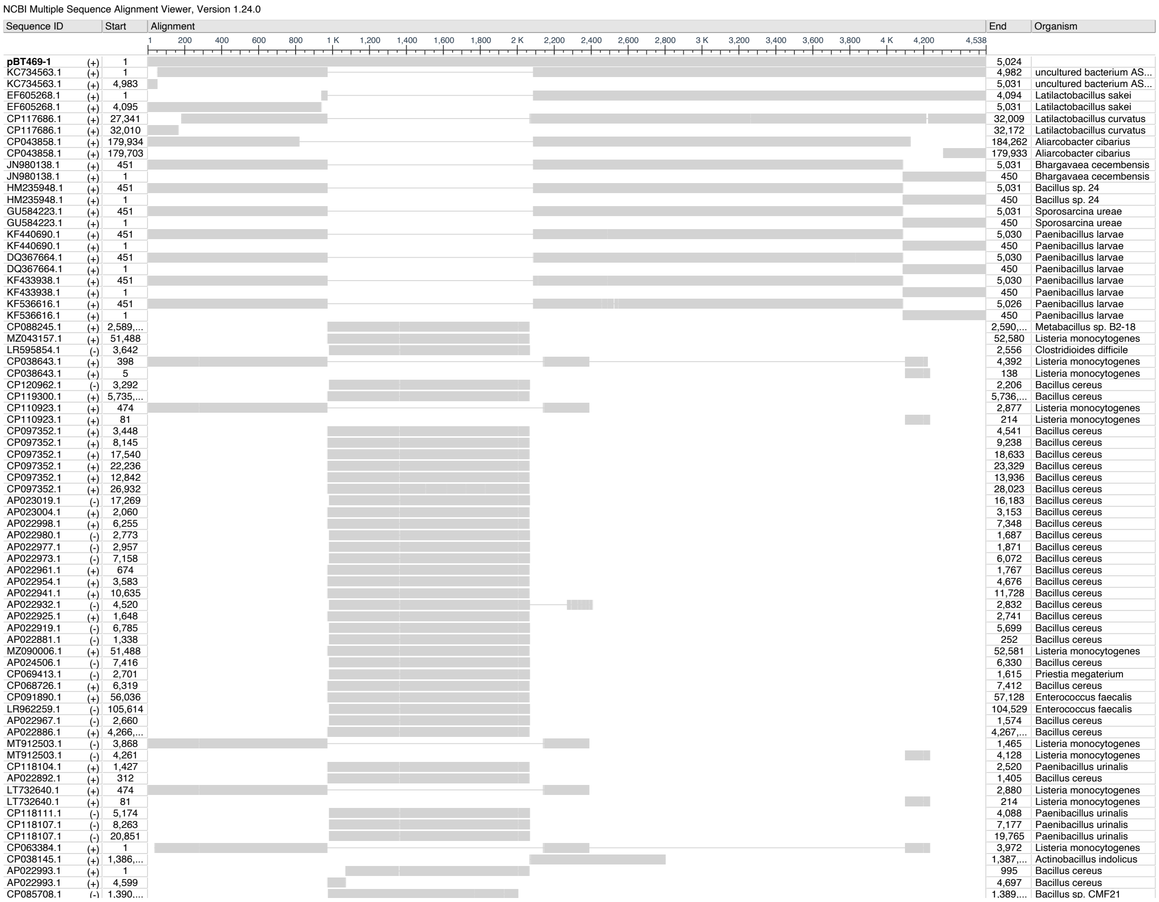

Figure S2. BLASTn result (50 best hits) of the pBT469-1 sequence (in bold) against the nr/nt database. Gray bars represent homologous regions.

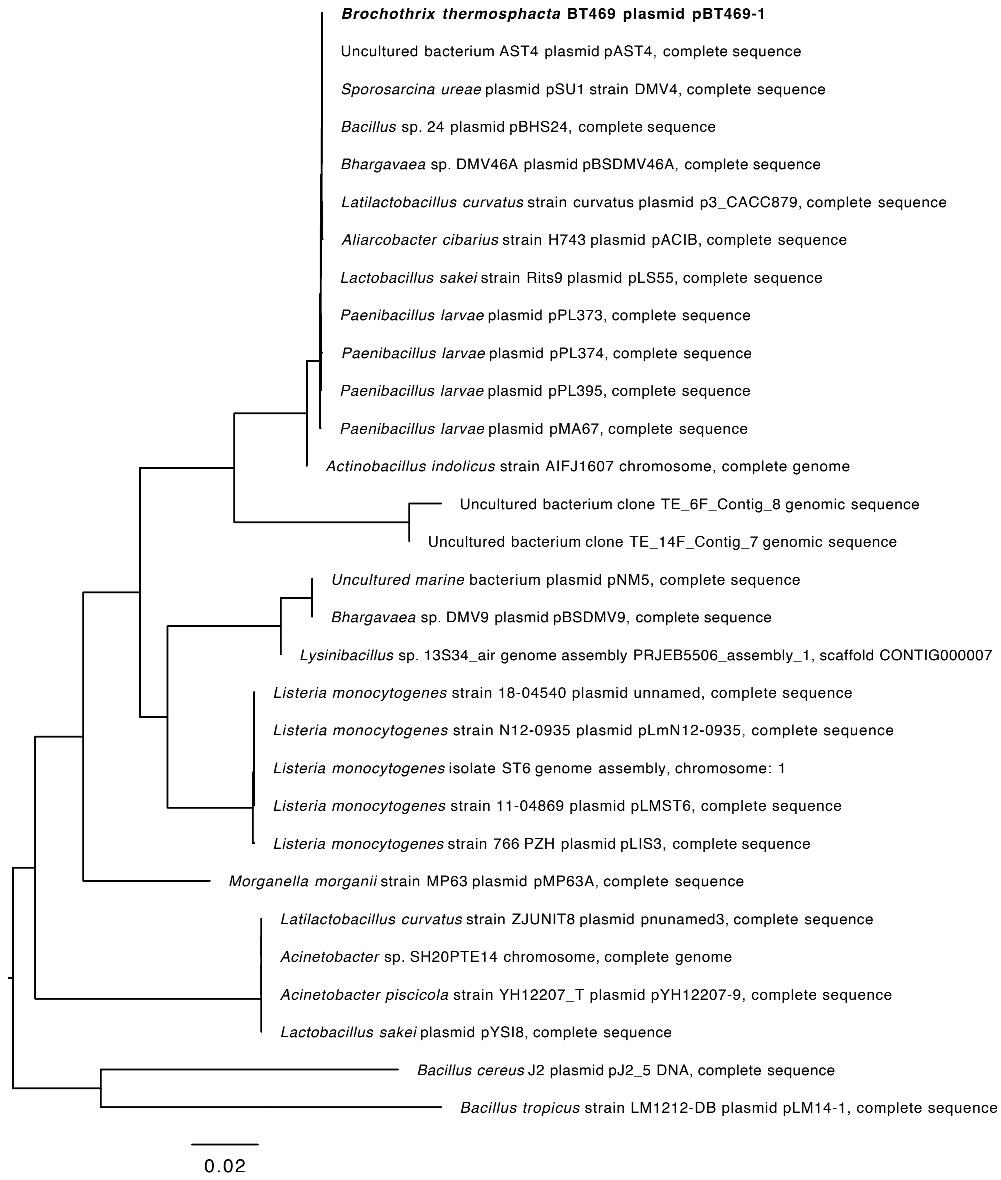

Figure S3. Phylogenetic tree generated with BLASTn from the NCBI server. The sequence of plasmid pBT469-1 was used against the nr/nt database.

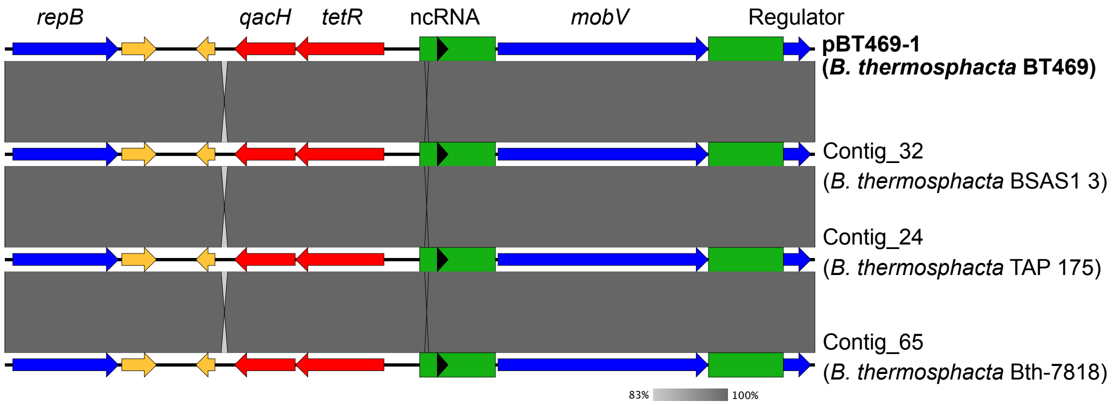

Figure S4. Alignment of the pBT469-1 sequence against the homologous sequences in the wgs database for *B. thermosphacta*. Homologous regions between sequences are shown in gray. The red, yellow, and blue arrows, respectively, represent the genes coding for proteins involved in resistance to antibiotics, with a hypothetical function, or for another function (for example the maintenance of the plasmid or its mobilization). Green and black arrows represent regulatory regions and non-coding RNAs (ncRNAs), respectively.

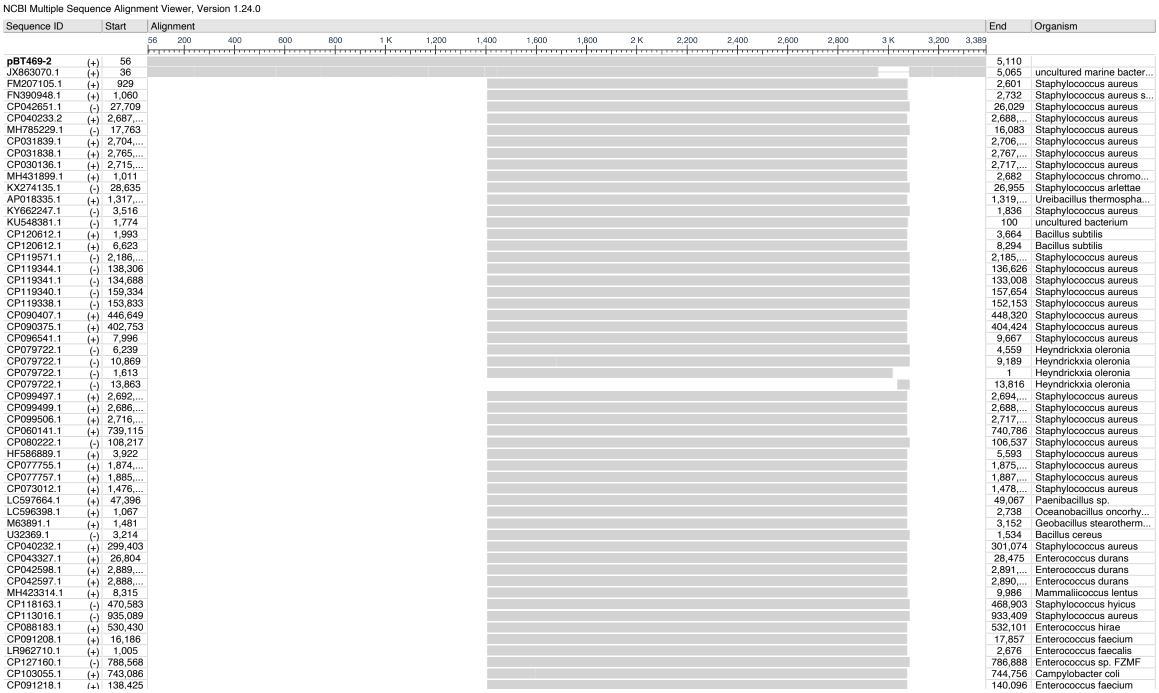

Figure S5. BLASTn result (50 best hits) of the pBT469-2 sequence (in bold) against the nr/nt database. Gray bars represent homologous regions.

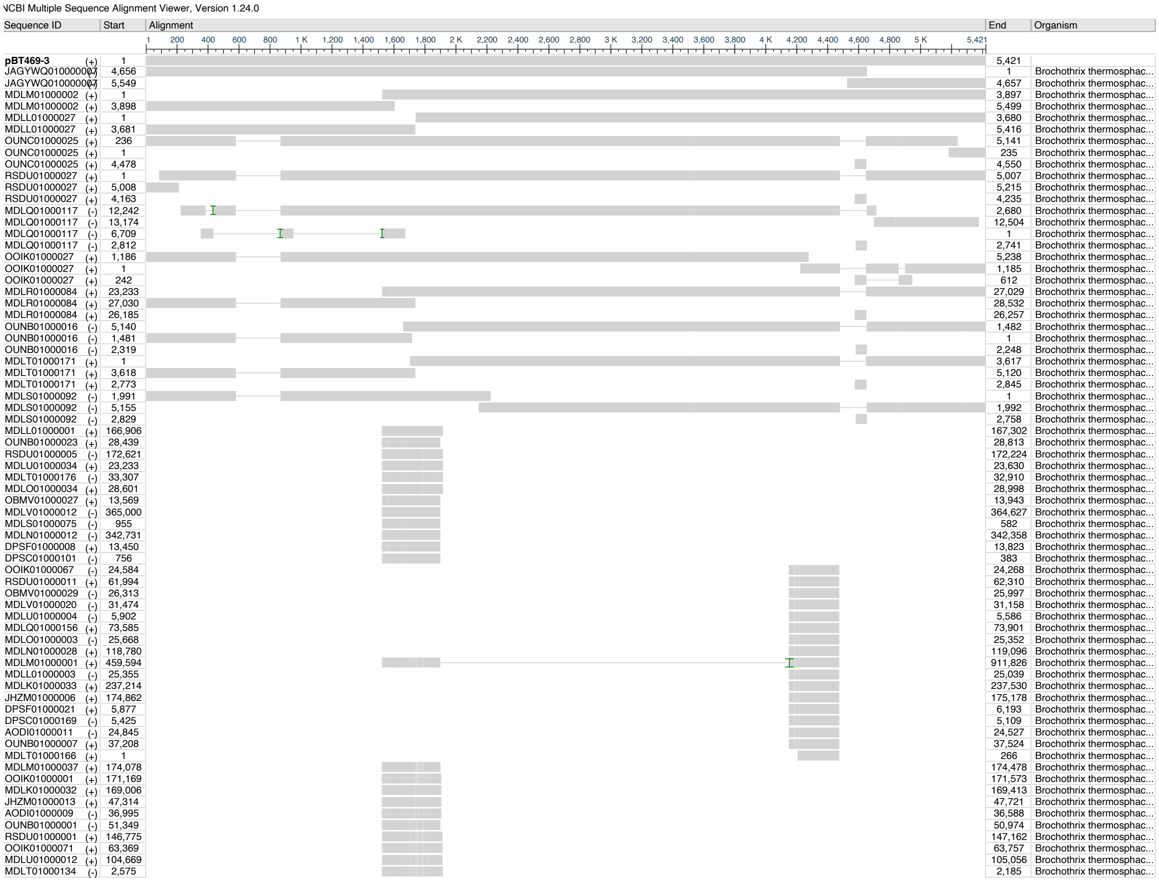

Figure S6. BLASTn result (50 best hits) of the pBT469-3 sequence (in bold) against the wgs database (for *B. thermosphacta*). Gray bars represent homologous regions.
